# True Congenital Pancreatic Cyst: A Rare Entity

**DOI:** 10.7759/cureus.3318

**Published:** 2018-09-17

**Authors:** Mina'a Shahid, Zarmina Javed, Muhammad Usman, Salma Iltaf

**Affiliations:** 1 Miscellaneous, Shifa International Hospital, Islamabad, PAK; 2 Internal Medicine, Shifa International Hospital, Islamabad, PAK; 3 General Surgery, Cleveland Clinic Foundation, Clevaland, USA; 4 Pathology, Shifa International Hospital, Islamabad, PAK

**Keywords:** pancreatic cyst, congenital, cystoduodenostomy, cystojejunostomy

## Abstract

Pancreatic cysts are common in adults but rarely seen in children. Out of those seen in children, the majority are pseudocysts. This article presents a unique case of a child diagnosed with a true congenital pancreatic cyst which was ultimately excised completely.

## Introduction

Pancreatic cysts are commonly found in adults, but extremely rare in infants and children. Cysts can be true cysts or pseudocysts. A true cyst has an epithelial lining which secretes fluid, whereas pseudocysts have a fibro-inflammatory lining [1]. The fibro-inflammatory lining is made up of fibrous and granulation tissue but lacks an epithelium. In children, as compared to true pancreatic cysts, pseudocysts are more commonly seen [2].

During literature review, multiple articles were found on congenital pancreatic pseudocysts but very few on true congenital pancreatic cysts. This article reports a pediatric case of a true congenital pancreatic cyst.

## Case presentation

A 13-year-old child presented to a tertiary care hospital with abdominal pain, nausea, and vomiting. The severity of the pain was recorded as 5/10 when mild and 8/10 when severe. He experienced nausea and vomiting after every meal.

On physical examination, the patient had normal vitals. An abdominal exam showed tenderness in the epigastric region but no visceromegaly, and bowel sounds were normal.

Previously, at the age of one year, he presented to a primary care hospital with episodic epigastric pain. An abdominal ultrasound was performed which showed two cystic structures in the left lumbar region. This was followed by a computed tomography (CT) scan at the time, which showed two cysts in the body and tail of the pancreas. The body and tail of the pancreas were not visualized separately. Stranding of adjacent peri-pancreatic soft tissue and minimal thickening of the adjacent duodenum were also seen. A diagnosis of congenital pancreatic cyst was established.

In the tertiary care hospital, the child was investigated further. Peripheral blood count revealed a white cell count of 12600/micro litre, with slightly raised lymphocytes (49%). The red cell indices were partially low (mean corpuscular volume (MCV) = 76.1 femto litre, mean corpuscular hemoglobin (MCH) = 25.1 picogram) but hemoglobin was in the normal range. The rest of the routine lab tests were normal.

**Figure 1:**
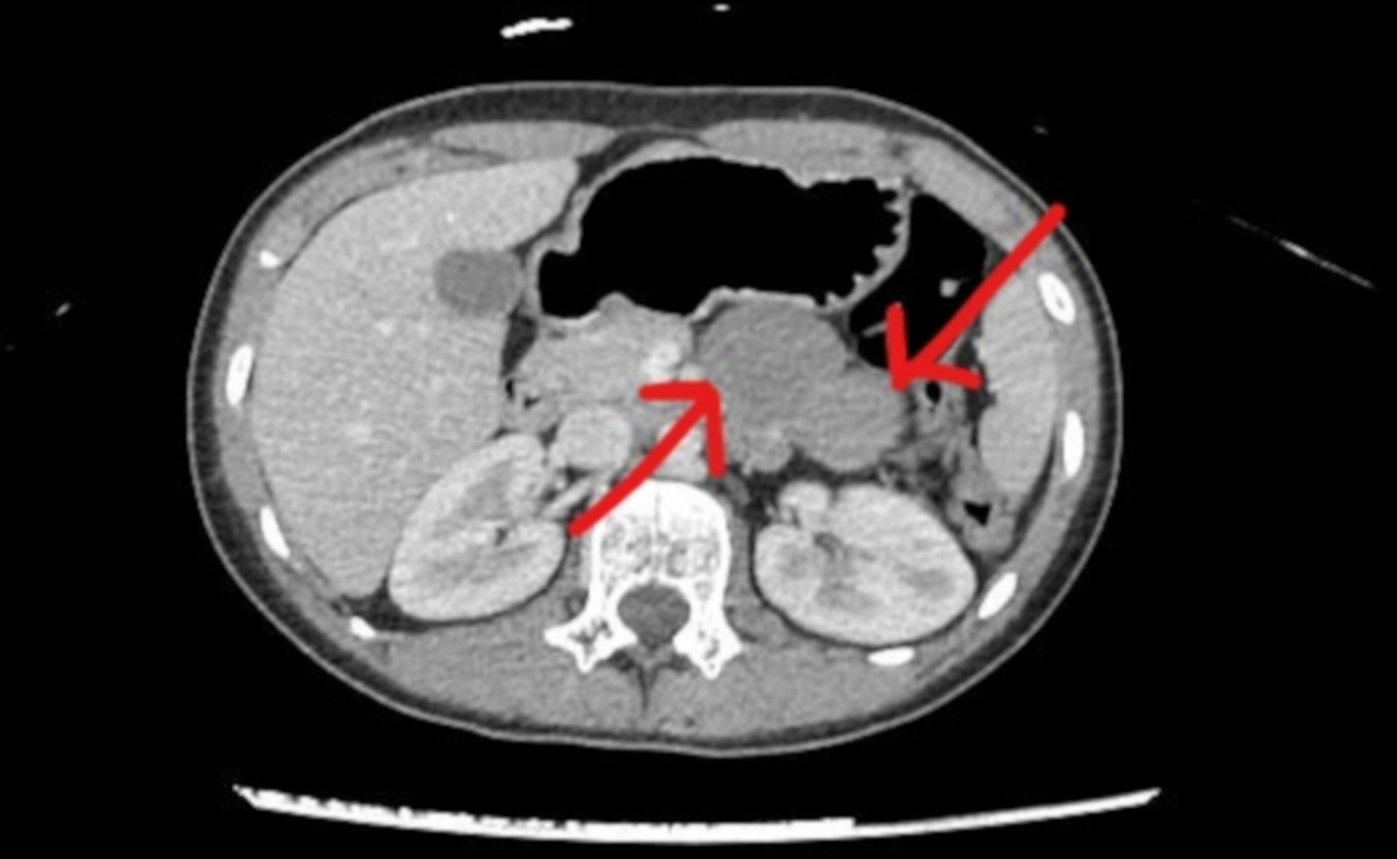
Computed tomography (CT) scan of the abdomen, with contrast, showing a bi-lobed soft tissue density lesion (arrows), inseparable from the pancreas

A CT scan was repeated in the tertiary care hospital. It showed a bi-lobed soft tissue density lesion measuring 7.2 × 5.8 × 2.8 cm (cranio-caudal×transverse×antero-posterior (CC×TR×AP)), located between the stomach and the pancreas. It was seen to be inseparable from the pancreas and projecting into the mesentery. Mild hepatomegaly was also reported (Figure 1). This CT scan report confirmed the diagnosis of a pancreatic cyst.

Given the patient's worsening symptoms of severe pain, nausea, and vomiting, and the CT findings, it was decided to proceed with an exploratory laparotomy. The cyst was excised successfully and a sample was sent for histopathology. Post-surgery, the patient remained stable and was discharged after a few days.

**Figure 2:**
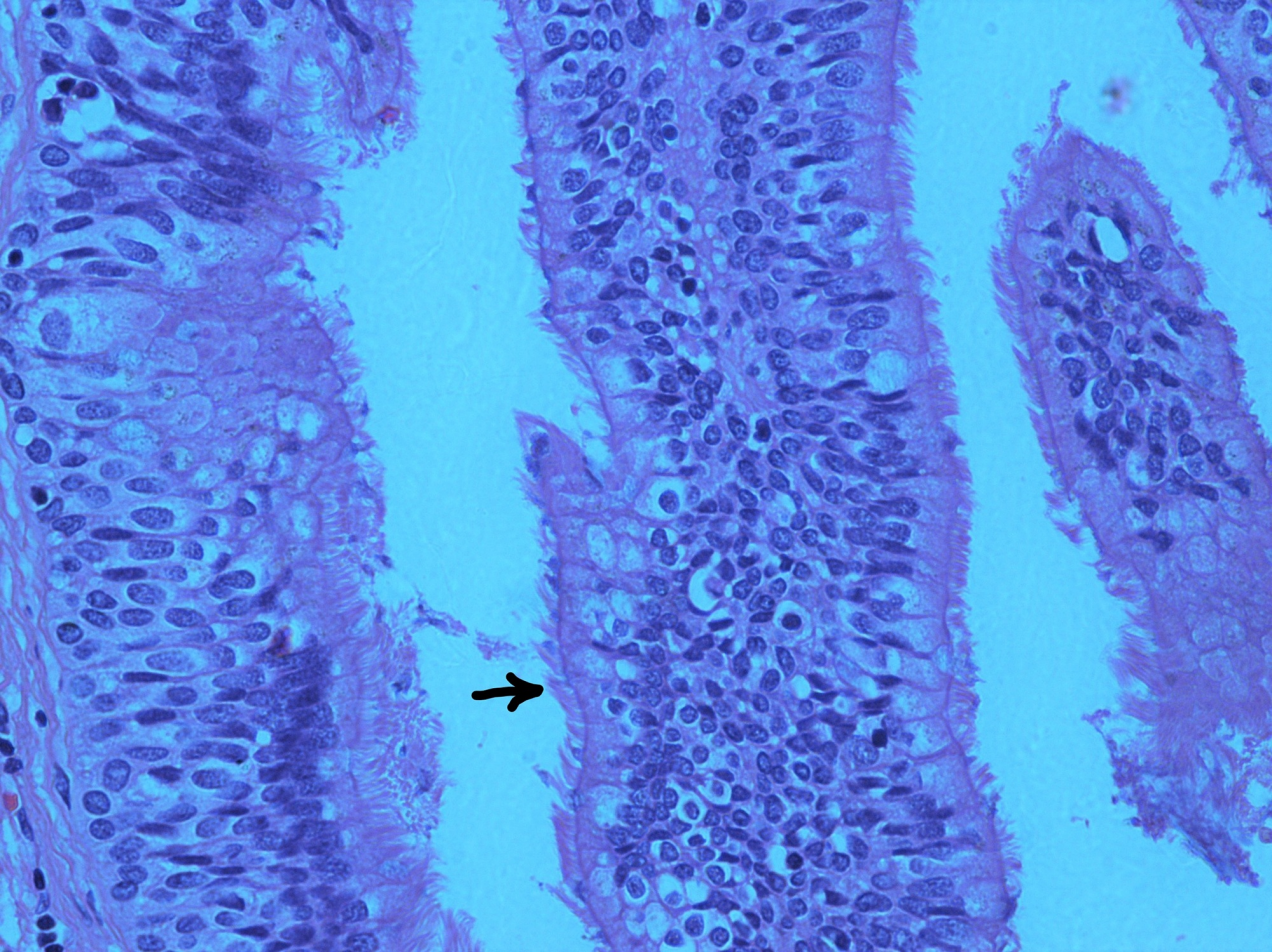
Haematoxylin and eosin (H&E) section of the pancreatic cyst showing respiratory epithelium (ciliated pseudostratified columnar epithelium); the arrow points to the cilia (×40)

**Figure 3:**
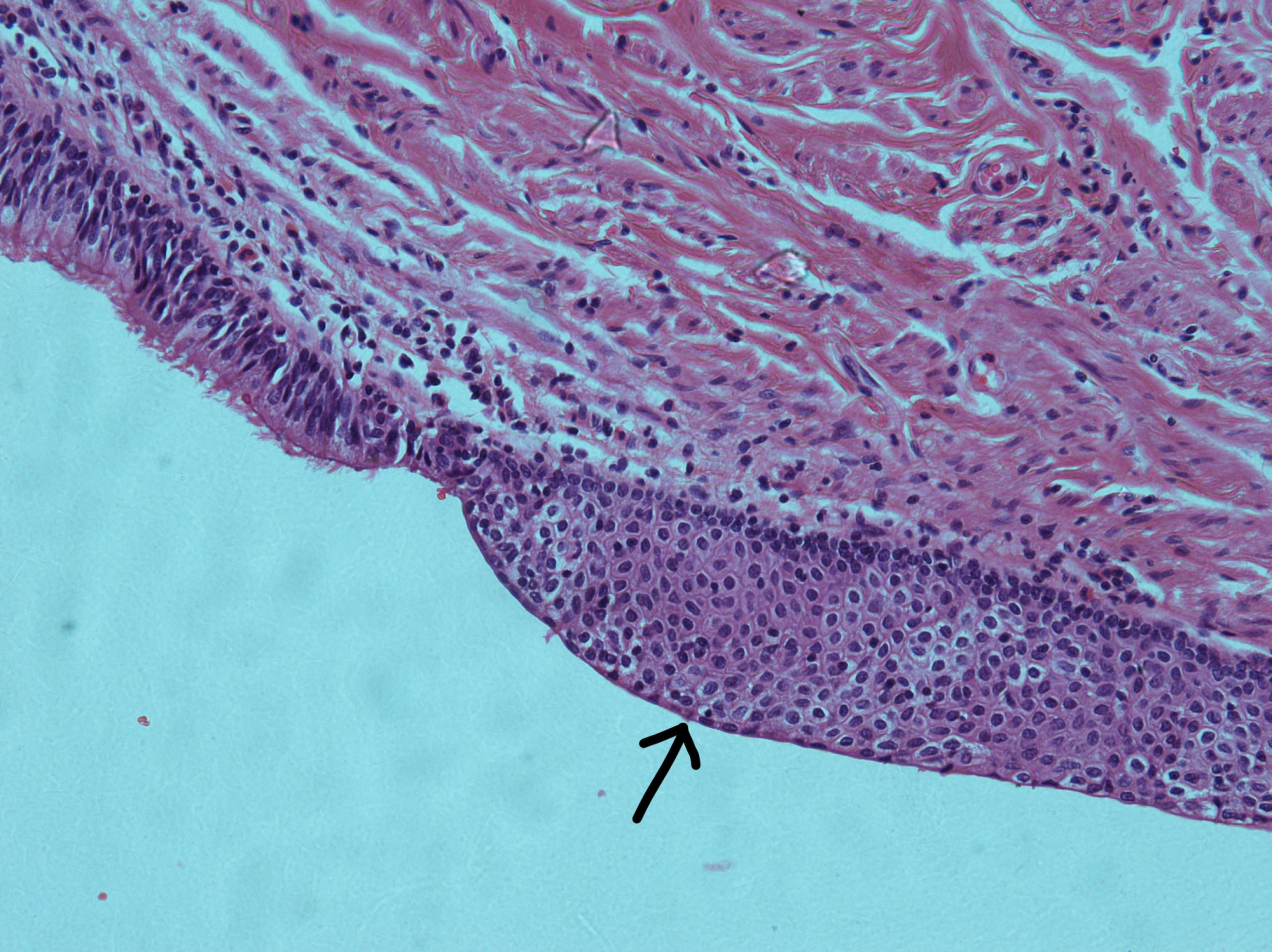
Haematoxylin and eosin (H&E) section of the pancreatic cyst demonstrating squamous metaplasia (arrow) (×20)

**Figure 4:**
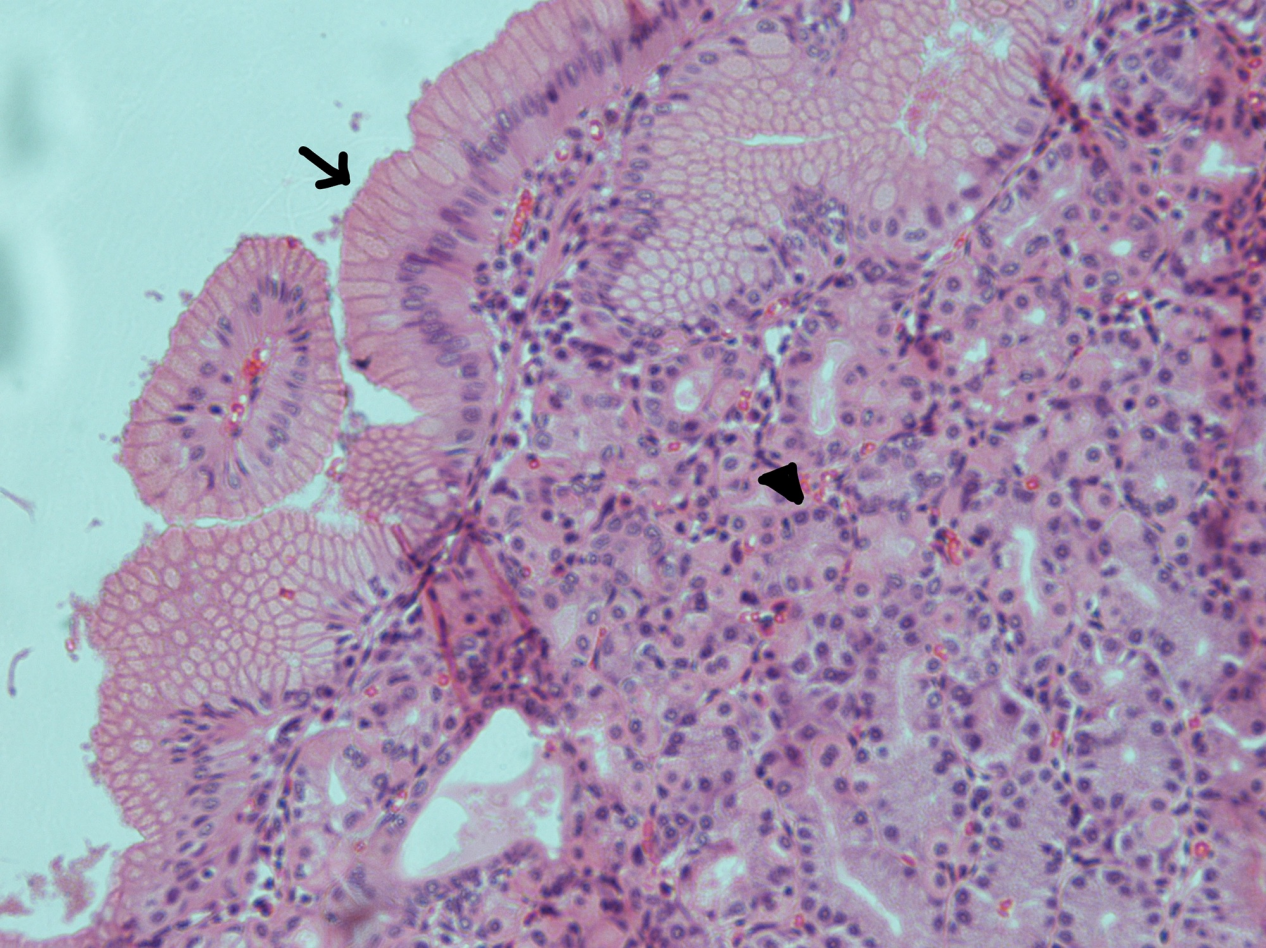
Haematoxylin and eosin (H&E) section of the pancreatic cyst showing gastric epithelial lining; the arrow shows columnar epithelium and the arrowhead points to a gastric gland (×20)

The histopathology report confirmed a true congenital pancreatic cyst. Grossly, the specimen consisted of a cystic structure measuring 8.0 × 3.5 × 3.5 cm. Opening the outer surface revealed a bi-lobed cyst separated by fibrous septae. It contained a thick brown material. Microscopically, the cyst walls were seen to be lined by respiratory epithelium with areas of squamous metaplasia (Figures 2-3). Certain sections also had areas of gastric epithelial lining with the underlying stroma showing irregularly arranged smooth muscle layer (Figure 4). There was no evidence of malignancy in the sample examined.

## Discussion

Pancreatic cysts are extremely rare in the pediatric population [3]. Until the 1970s, cystic lesions of the pancreas were classified as either mucinous or serous neoplasms. However, in the 1980s, due to the introduction of new imaging techniques, multiple new entities of pancreatic cysts were revealed [4]. Currently, pancreatic cysts are majorly subdivided into the following categories: congenital, retention, duplication, pseudocysts, neoplastic, and parasitic cysts. True congenital cysts are the rarest among these [3].

The exact etiology of congenital pancreatic cysts is unknown but they are considered to be a consequence of persistence or failure of embryonic pancreatic ducts to regress. When they get obstructed, fluid-filled cystic lesions form [5]. The origin of congenital pancreatic cysts varies; 62% are localized to the tail or neck, as was observed in our patient, and 32% to the head of the pancreas [3].

Cystic lesions of the pancreas are small and asymptomatic, often detected incidentally [2]. They may present with abdominal pain, abdominal distention, vomiting, jaundice or pancreatitis secondary to the pressure on adjacent structures. Pancreatic cysts can be isolated, or associated with asphyxiating thoracic dysplasia (Jeune syndrome), von Hippel-Lindau disease, Beckwith-Wiedemann syndrome, Ivemark syndrome, hemihypertrophy, renal tubular ectasia, anorectal malformations, and polycystic kidneys [5]. Although our patient suffered the symptoms of abdominal pain, abdominal distention, and vomiting, he did not endure any of these disease conditions, which could have resulted in earlier excision of the cyst.

True congenital pancreatic cysts are difficult to diagnose pre-operatively. Ultrasonography is the most rapid and consistent way to differentiate between solid and cystic structures. However, CT scan or magnetic resonance imaging (MRI) is essential for proper localization of the cyst. A CT scan gives a more accurate picture as to the origin of the cyst whereas MRI is better at establishing the extension of the cyst into surrounding structures [6]. Nevertheless, it is very difficult to differentiate between different types of congenital pancreatic cysts on the basis of imaging. Endoscopic retrograde cholangiopancreatography (ERCP) can portray any communication of the cyst with pancreatic duct or biliary tree, but being an invasive procedure, it is not considered the best diagnostic modality for pancreatic cysts [2]. Endoscopic ultrasound (EUS) is being used in adults to further evaluate pancreatic cysts. It provides better images due to the closeness of the transducer to the area of interest, helping to better visualize the cyst. EUS also helps with taking samples of cystic fluid for enzymes and tumor markers. This helps differentiate between various types of cysts and in ruling out malignancies [7].

The preferred treatment for congenital pancreatic cysts is complete surgical excision, as was performed in our patient. The cysts located in the body and tail of the pancreas are excised completely along with distal pancreatectomy while those located in the head are difficult to approach and drained internally through cystoduodenostomy or Roux-en-Y cystojejunostomy [3].

## Conclusions

Pancreatic cysts are rare in infants and children, especially true congenital pancreatic cysts. They are mostly discovered incidentally. Hence, in infants and children presenting with abdominal masses or something as simple as epigastric pain and/or vomiting, congenital pancreatic cysts should not be disregarded.
